# Genome-wide identification, characterization, and expression analysis of MIPS family genes in legume species

**DOI:** 10.1186/s12864-023-09937-7

**Published:** 2024-01-23

**Authors:** Feba Jacob, Rasmieh Hamid, Zahra Ghorbanzadeh, Ravisankar Valsalan, Lavale Shivaji Ajinath, Deepu Mathew

**Affiliations:** 1 Centre for Plant Biotechnology and Molecular Biology, Kerala Agricultural University, Thrissur, India; 2 Department of Plant Breeding, Cotton Research Institute of Iran (CRII), Agricultural Research, Education and Extension Organization (AREEO), Gorgan, Iran; 3 Department of Systems Biology, Agricultural Biotechnology Research Institute of Iran (ABRII), Agricultural Research, Education and Extension Organization (AREEO), Karaj, Iran

**Keywords:** Antinutritional factor, Biofortification, Fabaceae, Genome annotation, Pulses, Expression analysis

## Abstract

**Background:**

Evolutionarily conserved in plants, the enzyme D-myo-inositol-3-phosphate synthase (*MIPS*; EC 5.5.1.4) regulates the initial, rate-limiting reaction in the phytic acid biosynthetic pathway. They are reported to be transcriptional regulators involved in various physiological functions in the plants, growth, and biotic/abiotic stress responses. Even though the genomes of most legumes are fully sequenced and available, an all-inclusive study of the *MIPS* family members in legumes is still ongoing.

**Results:**

We found 24 *MIPS* genes in ten legumes: *Arachis hypogea*, *Cicer arietinum*, *Cajanus cajan*, *Glycine max*, *Lablab purpureus*, *Medicago truncatula*, *Pisum sativum*, *Phaseolus vulgaris*, *Trifolium pratense* and *Vigna unguiculata*. The total number of *MIPS* genes found in each species ranged from two to three. The *MIPS* genes were classified into five clades based on their evolutionary relationships with *Arabidopsis* genes. The structural patterns of intron/exon and the protein motifs that were conserved in each gene were highly group-specific. In legumes, *MIPS* genes were inconsistently distributed across their genomes. A comparison of genomes and gene sequences showed that this family was subjected to purifying selection and the gene expansion in *MIPS* family in legumes was mainly caused by segmental duplication. Through quantitative PCR, expression patterns of *MIPS* in response to various abiotic stresses, in the vegetative tissues of various legumes were studied. Expression pattern shows that *MIPS* genes control the development and differentiation of various organs, and have significant responses to salinity and drought stress.

**Conclusion:**

The *MIPS* genes in the genomes of legumes have been identified, characterized and their expression was analysed. The findings pave way for understanding their molecular functions and evolution, and lead to identify the putative *MIPS* genes associated with different cell and tissue development.

**Supplementary Information:**

The online version contains supplementary material available at 10.1186/s12864-023-09937-7.

## Background

Myo-inositol-1-phosphate synthase (*MIPS*; EC 5.5.1.4), is a key, rate-limiting enzyme for the initial reaction in the lipid-independent pathway of phytic acid biosynthesis, wherein D-glucose-6-phosphate (G6P) is converted to inositol phosphate, which further is dephosphorylated to Myo-inositol (Ins), by Myo-inositol monophosphatase (*IMP*) [1]. *MIPS* genes are evolutionarily conserved and found in various species, including higher eukaryotes viz. humans and higher plants, as well as cyanobacteria, eubacteria, and archaea [2, 3]. It has also been found in a number of plants, including *Arabidopsis thaliana* [4], *Oryza sativa* [5], kiwi fruit [6], *Glycine max* [7, 8], *Phaseolus vulgaris* [9], and *Sesamum indicum* [10]. The eukaryotic *MIPS* family is homogeneous and yet distinct from the bacterial MIPS proteins in their sequence [11, 12]. This could be explained by the eukaryotic *MIPS* genes’ monophyletic origin. The *MIPS* sequences (approximately 510 amino acids), in all the eukaryotes, exhibit considerable conservation at the nucleotide level [13], signifying the crucial functions that *MIPS* play in various biological processes such as seed germination and embryogenesis. The *MIPS* proteins have four amino acid domains, GWGGNNG (domain 1), LWTANTERY (domain 2), NGSPQNTFVPGL (domain 3) and SYNHLGNNDG (domain 4) that are highly conserved [14]. These domains perform crucial roles in *MIPS* protein binding for the catalysis of enzymatic processes and thus regulate *MIPS* activities [15].

The *MIPS* regulates the biosynthesis of Ins and its derivatives such as phosphoinositide phosphates (PtdInsPs), Ins polyphosphates (IPs), and phospholipid phosphatidylinositol (PtdIns), which play important and varied roles in cell division, plant growth, organ development as well as biotic and abiotic stress responses [16]. This gene was reported for the first time in *Archaeoglobus fulgidus* and was observed to function at high temperatures [17]. Earlier studies have shown that three *MIPS* genes play important roles in *Arabidopsis* embryo development [18]. Ma et al. [19] showed how the direct binding of two light-signaling protein molecules (FHY3 and FAR1) to the *MIPS*1 promoter stimulated inositol synthesis in response to light-induced oxidative stress. Surprisingly, Latrasse et al. [20] discovered that *MIPS1*, in addition to its role in enzymatic metabolic activity, is also transported to the nucleus where it regulates transcription. Donahue et al. discovered that *mips2* loss-of-function mutants of *Arabidopsis* are more susceptible to fungal, viral and bacterial diseases, whereas *mips1* mutants exhibited higher resistance to oomycete infections but also spontaneous cell death [1]. In addition, *mips1* mutants were also susceptible to severe light stress [21]. There were no major phenotypic differences in the single *mips* mutants, but the *mips1 mips2* double mutant as well as the *mips1 mips2 mips3* triple mutant were fatal to the embryo [22]. Significant suppression of plant growth was observed when the activity of *MIPS* was greatly reduced [15, 23]. Lower IP6 levels in the potatoes produced by RNA interference (RNAi)-mediated *MIPS* suppression has altered the leaf shape, promoted leaf senescence, and reduced tuber yield [24].

Legumes (Fabaceae) occupy about 27% of the primary production of the world and are grown on about 12% of the planet’s arable land [25, 26]. They help in fixing atmospheric nitrogen through the nitrogen-fixing bacteria that live symbiotically in their root nodules and thus are important nitrogen sources in crop systems [27]. Legumes are also gaining traction for the production of beneficial secondary metabolites that act as signaling molecules for attracting pollinators and seed dispersing animals and as chemical defenses against herbivores and microbes [28]. More efforts should be made to find and characterize genes associated with legume growth, development, and stress responses because they are among the world’s most important crops [29, 30]. Currently, knowledge about the *MIPS* gene family in Fabaceae and their contribution in legume growth and development is very little. A comparative analysis was carried out between the *MIPS* gene family in nonlegume (*Arabidopsis* and rice) and legume crops, to comprehend their roles in legumes, especially in seed germination, plant growth, and development, which included molecular traits, phylogenetic classifications, chromosome distribution, conserved motifs and synteny analysis. This is the first work on the *MIPS* gene family in legumes, which will support its functional research in related crops.

## Methods

### *MIPS* gene identification and characterization

The genomes and annotation files of 10 legume species including *Arachis hypogea* (peanut), *Cicer arietinum* (chickpea), *Cajanus cajan* (pigeon pea), *Glycine max* (soybean), *Lablab purpureus* (Dolichos bean/hyacinth bean), *Medicago truncatula* (barrel medic), *Pisum sativum* (pea), *Phaseolus vulgaris* (French bean/common bean), *Trifolium pratense* (clover), and *Vigna unguiculata* (cowpea) were retrieved from the Legumes database (https://plants.ensembl.org/index.html (accessed July 16, 2023)) and the Phytozome v13 database (http://www.phytozome.net (accessed July 16, 2023)) [31], and was used for the identification and annotation of *MIPS* genes (more information is available in Table 1). Further, three MIPS protein sequences (IDs: AT5G10170, AT4G39800 and AT2G22240) of *Arabidopsis* were obtained from the TAIR database (https://www.arabidopsis.org/). These sequences were used as query to perform BLASTp (e-value < 1 × 10^–5^) against various legumes to retrieve MIPS sequences. Thereafter, all the retrieved MIPS proteins were analyzed using the Pfam (http://pfam.xfam.org) [32], CDD (https://www.ncbi.nlm.nih.gov/cdd, [33] (accessed June 20, 2022)) and SMART (http://smart.embl-heidelberg.de (accessed June 20, 2022)) [34] databases, to validate the presence of the signature domains (Inos-1-P Synth; PF01658 and NAD_5 Binding; PF07994) in MIPS.

The DNA and protein characteristics of MIPS, including DNA sequence length, protein molecular weight, protein length, pI, and signal peptide prediction, were generated using ExPASy protparam (https://web.expasy.org/protparam, (accessed July 18, 2023)) [35], Phytozome v13 and UniProt (https://www.uniprot.org/ (accessed July 18, 2023)) [36].

### Multiple sequence alignment and phylogenetic analysis

To investigate the evolutionary relationships among the MIPS peptide sequences of legumes, multiple sequence alignment (MSA) of the amino acid sequences of all the ten legumes along with three MIPS proteins from *Arabidopsis*, was performed using CLUSTALW in MEGA version 11.0.13 [37]. The phylogenetic tree was constructed in MEGA using the following parameters: (1) Scope: all selected taxa, (2) phylogenetic test: bootstrap technique, (3) neighbour-joining (NJ) statistical approach, (4) 1000 bootstrap replicates, (5) Substitution types: amino acid, (6) Poisson model, (7) Uniform rates applied across all sites, (8) Homogeneous (same) ancestry pattern, and (9) Pairwise elimination for the treatment of gaps/missing. To visualize the phylogenetic tree, the iTOL45 web server (https://itol.embl.de/, [38]) was used.

### Gene structures, organization of motif, and prediction of domain

The Gene Structure Display Server (GSDS 2.0; http://gsds.cbi.pku.edu.cn/Gsds_abou.php) program was used to compare the CDS sequences with their corresponding genomic DNA sequences, to analyze the organization of the exonic and intronic regions as well as the untranslated region of the *MIPS* genes. The Motif Elicitation (MEME) program was employed to envisage the conserved motif with the parameters set to find out 20 motifs (http://meme-suit.org; [39]) and their structures were visualized using the TBtool software [40].

### Gene nomenclature, duplication, chromosomal location, and analysis of cis-regulatory elements

The *MIPS* genes reported in *Arabidopsis* were used to determine the nomenclature of the genes in each legume species. The closest orthologs to the *MIPS* genes of *Arabidopsis* in the evolutionary tree were used to determine the nomenclature of the genes in each legume species. From NCBI, the chromosome coordinates and the gene and protein IDs were obtained. The position of each gene on each chromosome was plotted on chromosome maps generated for each species using the MG2C tool (http://mg2c.iask.in). For the analysis of the cis-regulatory elements (CREs) of *MIPS*, the sequences upstream (2000 bp) to the start codon were also retrieved from the corresponding database. The PlantCARE online server (https://bio.tools/plantcare) was used to identify the CREs in *MIPS* genes [41].

### Nuclear localization signals, GO analysis and subcellular localization

To predict the *MIPS* NLS, the online tool cNLS Mapper (https://nls-mapper.iab.keio.ac.jp) [42] was used. The PANNZER server (Protein ANNotation with Z-scoRE) (http://ekhidna2.biocenter.helsinki.fi/sanspanz) [43] was employed to get the gene ontology (GO) annotation of the legume MIPS using their respective gene ID. Further, for predicting subcellular localization of legume MIPS, online resource WoLF PSORT II (https://www.genscript.com/wolf-psort.html?src=leftbar) [44] was used.

### Plant materials and optimum growing conditions

Dolichos bean (*Dolichos lablab* L. cv. Hima) seeds were grown at ambient temperature (25 °C) in the greenhouse with a light/dark cycle of 14 h/10 h in pots filled with sterile mixtures (1:1) of agro-peat and vermiculite. The tissue-specific gene expression was analysed, for which the bud, leaf and root tissues were collected at the flowering stage (three months). All tissues were collected in three biological replicates and kept at -80 °C until used. PEG-6000 (20%) was used to induce drought stress in dolichos bean plants.

### Isolation of total RNA, cDNA preparation and RT-qPCR validation

Purelink® Plant RNA purification reagent was used to isolate the total RNA, from 100 mg of tissue, following the manufacturer’s guidelines. From this, 5.0 µg of total RNA was treated using the RNase-Free DNase Set (Qiagen) for removing the contaminating DNA. Using nano-volume spectrophotometer (NanoDrop Technologies) and Bioanalyzer (Agilent Technologies), the quantity and quality of the RNA samples were evaluated. Only RNA samples with the following characteristics were selected for analysis: 260/230 absorbance ratios between 2.0 and 2.4, 260/280 ratios between 1.9 and 2.1, and an RNA integrity number (RIN) larger than 7.0. The RevertAid First Strand cDNA Synthesis Kit (ThermoScientific) was used to create cDNA from the total RNA, following the manufacturer’s instructions. A reaction mix of 10 µL containing 2X SyBr Green Master mix (5.0 µL) (Applied Biosystems™), 10 mM of each primer, and cDNA (100 ng) was used to perform the real-time PCR assay on the Applied Biosystems™ qPCR, and the temperature profile for the reaction was 95 °C (2 min), 40 cycles of 95 °C (15 s) and 60 °C for 1 min. Supplementary Table S1 provides the details of the primers used in this study. Three each of biological and technical replicates were made in each reaction. The 2^−△△Ct^ technique was followed to analyze the data and calculate the relative expression.

## Results

### Identification and characterization of *MIPS* genes from legume genomes

To find the genes encoding proteins containing the MIPS domain, genome-wide searches were conducted by BLASTp. Further, HMMER was used for a thorough search and identification of *MIPS* genes in each of the legume proteomes. Results obtained were confirmed for the presence of the MIPS domain using Pfam, SMART, and NCBI Conserved Domains Database. A few protein sequences that were incomplete in the N-terminal region of the characteristic MIPS domain were excluded. In total, we found 24 potential *MIPS* genes and 47 different transcripts from 10 legume species (Supplementary Table S2). We gave each MIPS protein a unique name based on the *MIPS* genes reported in *Arabidopsis* and their chromosomal or scaffold position, using the species abbreviation as a prefix. Each legume had 2–3 *MIPS* genes, except soybean, which had four *MIPS* genes (Table 1, Supplementary Table S2).

**Table 1:** Comprehensive information of *MIPS* genes from ten legume species

**Gene name**	**Gene ID**	**Chromosome/ Location**^a^	**Gene length (bp)**	**Exon/ intron**	**Protein**		
					**L**	**pI**	**MW (KDa)**
CcMIPS1	KYP45217.1	1; 64,348..68643	4295	10/9	510	8.15	56.37143
CcMIPS2	KYP64058.1	1; 2,587,488..2590628	3140	10/9	510	5.7	56.52288
LpMIPS2	Labpu03g041840.1	3; 56,635,636..56640873	5237	10/9	510	5.55	56.41651
LpMIPS3	Labpu06g001800.1	6; 1,422,274..1426489	4215	10/9	510	5.24	56.46354
CaMIPS1	Ca_09586	6; 7,894,342..7898451	4109	10/9	510	5.51	56.17
CaMIPS2	Ca_12683	5; 43,684,761..43687739	2978	10/9	510	5.37	56.17
AhMIPS3	arahy.23HYFJ.2	1; 127,510,321..127515306	4985	9/8	510	5.47	56.488
AhMIPS1	arahy.96MT2N.1	1; 14,202,165..14206221	4056	10/9	510	5.47	56.61474
AhMIPS2	arahy.NNJC7T.1	1; 21,695,121..21699129	4008	10/9	510	5.47	56.62877
GmMIPS3	Glyma.05G180600	5; 36,870,767..36875196	4429	10/9	510	5.55	56.42054
GmMIPS4	Glyma.11G238800.1	11; 33,319,225..33322244	3019	9/8	509	5.31	56.47567
GmMIPS1	Glyma.08G138200	8; 10,592,506..10597043	4537	10/9	526	5.74	58.90235
GmMIPS2	Glyma.18G018600	18; 1,365,144..1368075	2931	10/9	510	5.37	56.44452
MtMIPS1	Medtr3g087590	3; 39,697,586..39701455	3869	10/9	510	5.44	56.58172
MtMIPS2	Medtr8g091320.1	8; 38,077,427..38081222	3795	10/9	510	5.46	56.47743
PvMIPS1	Phvul.001G251000.1	1; 50,146,237..50149206	2969	10/9	510	5.75	56.44763
PvMIPS2	Phvul.002G261700	2; 43,366,988..43371400	4412	10/9	510	5.42	56.47854
TpMIPS1	Tp57577_TGAC_v2_gene1741	3; 8895..12131	3236	9/8	414	5.31	45.83665
TpMIPS2	Tp57577_TGAC_v2_gene22605	8; 7,181,230..7185372	4142	1/0	126	6.7	14.07147
TpMIPS3	Tp57577_TGAC_v2_gene1515	11 6329..9176	2937	5/4	199	8.52	22.33476
VuMIPS4	Vigun03g073900	3; 6,093,230..6097413	4183	10/9	510	5.48	56.4385
VuMIPS1	Vigun01g235400	1; 40,693,586..40696720	3134	10/9	510	5.39	56.4205
PsMIPS2	KAI5406008.1	5; 204,536,777..204540143	3366	5/4	510	5.44	56.55
PsMIPS1	KAI5384408.1	7; 96,888,617..96,891,115	2498	9/8	529	5.39	58.63

^a^Start position; *E* Exons, *L* Length (aa), *NA* Not available

The basic parameters of the identified proteins such as protein length, isoelectric point, molecular weight, subcellular localization, and functional annotation are presented in Table 1 and Supplementary Table S2. The length of *MIPS* genes in different legumes ranged from 2498 (*PsMIPS1*) to 5237 bp (*LpMIPS2*), and their average was 3770 bp. The length of MIPS protein sequences showed a bimodal distribution, ranging from 126 aa (TpMIPS4) to 529 aa (PsMIPS1) and the average was 479 aa. The length of the MIPS proteins fell into any one of two categories: (i) 509–529 amino acid residues for 21 MIPS proteins or (ii) 129–414 amino acid residues for three MIPS proteins. The predicted molecular weight of the proteins ranged from 14.07 (TpMIPS2) to 58.90 kDa (GmMIPS1), and the average molecular weight was 53.01 kDa. The range of isoelectric points of the proteins was 5.24 to 8.52.

**Fig. 1:**
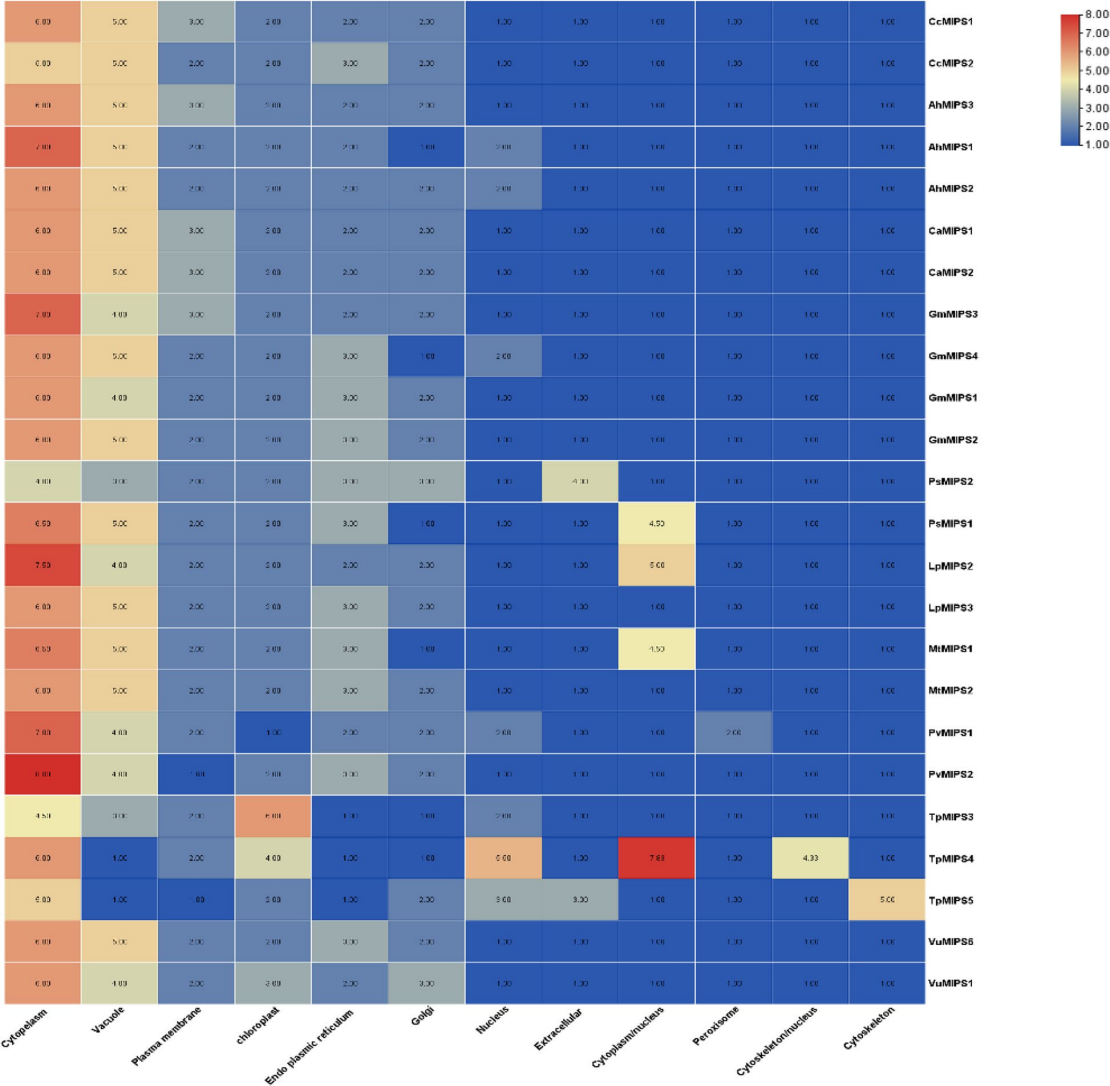
The MIPS genes’ subcellular localization. The heatmap was created using WoLF PSORT data, and the colour scale depicts the abundance of organelles. Higher expression levels were shown in red, while lower levels were shown in blue

The analysis of subcellular locations had shown that the proteins are localized in multiple organelles and most were located in the cytoplasm, plasma membrane, and vacuole (Fig. 1).

### Phylogenetic analysis and classification of *MIPS* genes

**Fig. 2:**
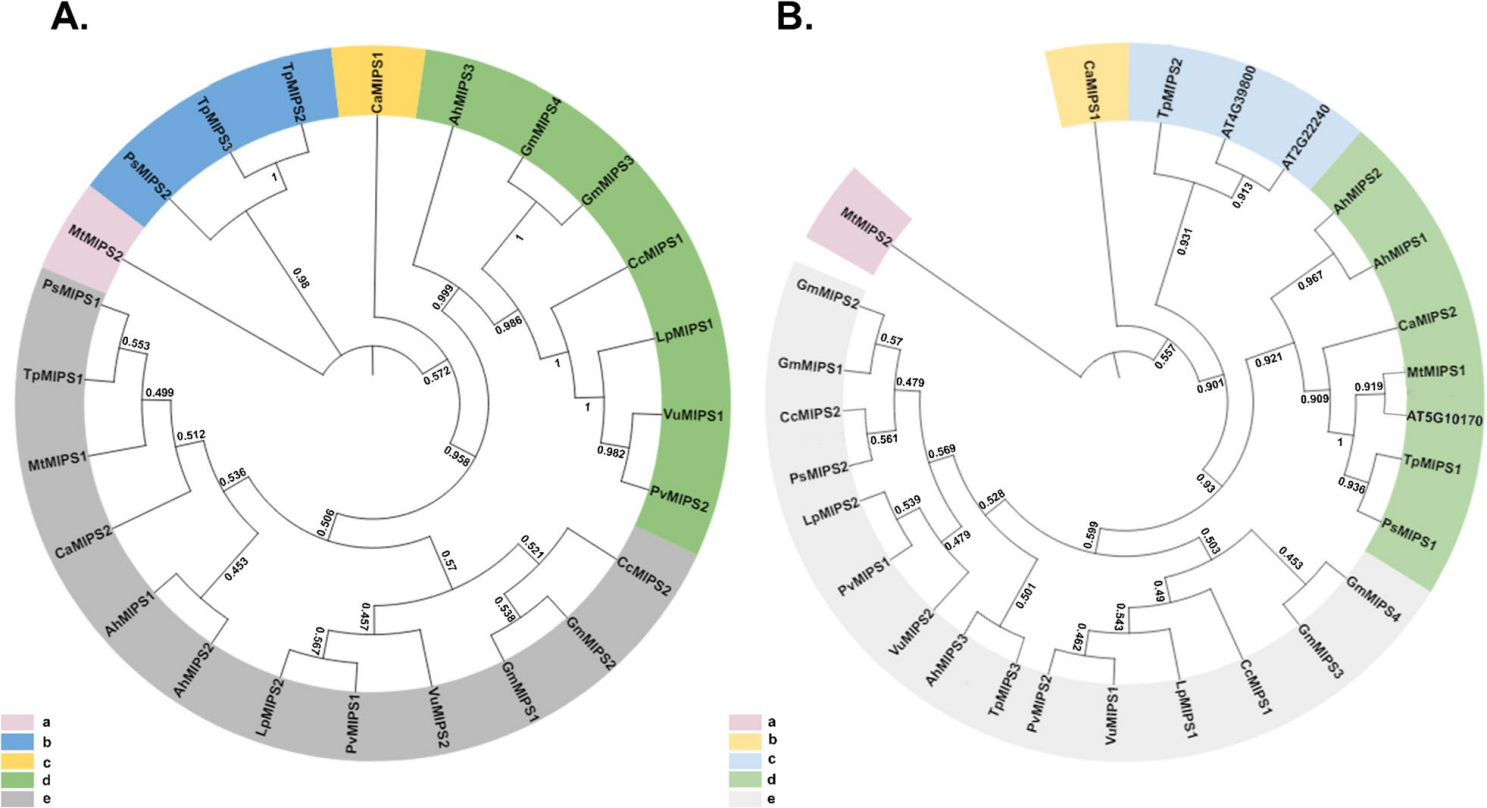
Two unrooted phylogenetic trees of *MIPS* genes were constructed by MEGA11: the evolutionary tree of the *MIPS* family was constructed using the neighbor-joining method, and the interspecific evolutionary tree of *MIPS* was constructed using the maximum likelihood method. **A** Phylogenetic tree of the *MIPS* family protein sequences in legume; **B** Phylogenetic relationships of 24 MIPS proteins from 10 legumes, and *Arabidopsis*

To comprehend the evolutionary relationships of *MIPS* genes, a multiple sequence alignment of 24 full-length peptide sequences from the legumes was performed and a phylogenetic tree was constructed using the neighbour-joining method (Fig. 2A). The MIPS proteins were thus classified into five clusters. Clusters B, C and D had bootstrap values > 80% and hence considered to have different phylogenetic lineages. The clusters A and E had bootstrap values less than 60%, having large variability among the members. To understand the phylogenetic relationship among the *MIPS* genes of legumes and *Arabidopsis*, an evolutionary tree was constructed with 24 sequences from legumes and three from *Arabidopsis*. The MIPS proteins from these species were distributed across almost all the branches. The evolutionary tree was divided into five subclades. Among them, subclades MIPS1 and MIPS2 have accommodated one sequence each, while MIPS4 had 15 and subclades MIPS3 and MIPS2 had seven and three sequences, respectively (Fig. 2B).

### Gene structure and conserved motif analysis

**Fig. 3:**
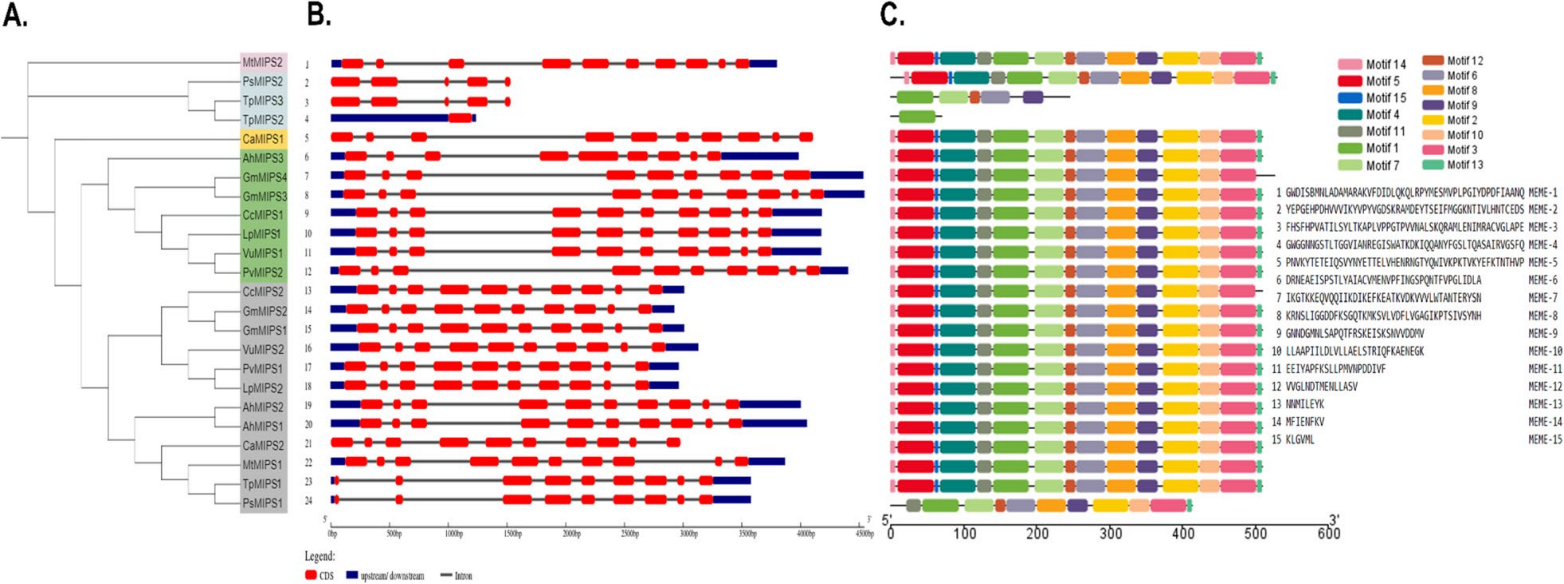
Genetic structure of the *MIPS* gene family in legume species **A** Phylogenetic tree of the *MIPS* gene family. **B** Motif pattern diagram of the *MIPS* gene family **C** Exon structure diagram of the *MIPS* gene family

Clear gene structure patterns were seen, which varied across groups but conserved within a group (Fig. 3A). The most common number of exons was ten, found in 17 *MIPS* genes (70.83%) (Fig. 3B). In total, there were 17 genes with nine introns each (70.83%), two with four introns (8.33%), four with eight introns (16.66%) and one gene (*TpMIPS*4) with no introns (4.16%). A, C, D and E group genes had eight to nine introns. On the contrary, genes in group B varied greatly with four to zero introns.

The validation of the classification of *MIPS* using MEME analysis [45] showed 10 conserved motifs that were statistically significant (E value lower than e^−100^) (Fig. 3C, Supplementary Table S3). The amino acid motif length ranged from 29 to 50. The consensus sequences of these conserved motifs and their amino acid lengths are shown in Supplementary Table S3. These ten motifs were observed in all legume genes except *CaMIPS1*, *TpMIPS2* and *TpMIPS3* and the pattern in which the motifs were arranged was also similar in 21 proteins (Fig. 3C). This suggests that *MIPS* is highly conserved in legumes.

### Location of *MIPS* on the chromosomes and gene duplication events

**Fig. 4:**
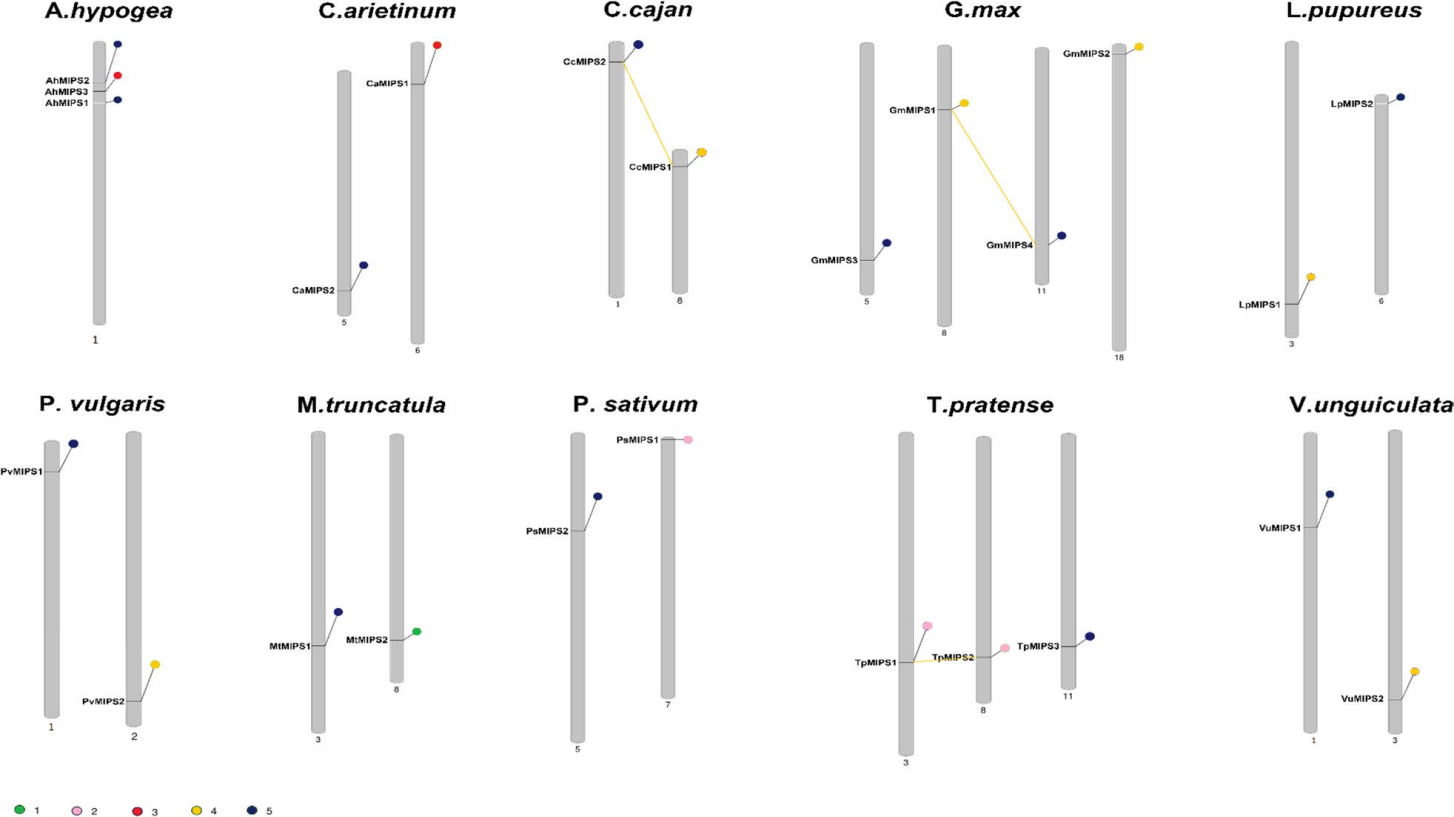
Chromosomal localization and gene duplication of *MIPS* genes in *Arachis hypogea*, *Cicer arietinum*, *Cajanus cajan*, *Glycine max*, *Lablab purpureus*, *Medicago truncatula*, *Pisum sativum*, *Phaseolus vulgaris*, *Trifolium pratense* and *Vigna unguiculata*., and tandem duplication of gene pairs during evolution is shown by lines

The distribution of the *MIPS* genes on the chromosomes of legumes was mapped with the programme MapInspect. We also examined the events of gene duplication in the *MIPS* gene family, and the output showed that *MIPS* gene pairs arose by tandem and segmental duplication and are connected by lines or shown in grey (Fig. 4). The legume chromosomes had an uneven distribution of *MIPS* genes. Tandem duplication contributed only slightly to the gene expansion of the *MIPS* family in legumes, but segmental duplication still had a significant impact. Seven pairs of genes evolved through tandem duplication and 17 pairs through segmental duplication were detected.

Out of all these legumes, it was observed that the gene duplication (including tandem and segmental duplication) of soybean was the most complicated, possibly due to hybridization [46, 47]. However, the gene expansion of the *MIPS* family in the lablab genome was only due to segmental duplication, whereas in the peanut genome, we did not find any indication of either tandem or segmental duplication of *MIPS* genes. Interestingly, many genes were duplicated both through tandem and segmental methods. For instance, ‘*TpMIPS1* and *TpMIPS2*’ and ‘*GmMIPS1* and *GMMIPS4*’ are gene pairs that denote tandem duplicates, while ‘*CcMIPS*2 and *CcMIPS*1’ is a gene pair resulting from chromosomal segmental duplication.

*MIPS* gene pairs that were segmentally duplicated are observed in all five groups (from A to E) of the phylogenetic tree, but such segmental duplication events are more prevalent between sister groups or within the same phylogenetic group. Fifteen of the 24 gene pairs that were segmentally duplicated had both the gene pairs in the same cluster and the remaining genes were mostly in pairs between two sister groups, such as ‘B and C’ or ‘D and E’. However, one exceptional gene pairing was between the *MtMIPS1* in group A in red clover, which paired with *TpMIPS*1 in group B and *TpMIPS*5 in group E. Tandem gene duplication was observed only in phylogenetic groups D and E, where the genes were paired only within the same phylogenetic group. Furthermore, the sequence similarity of all the tandemly duplicated gene pairs was comparatively higher (> 75%). Among them, “*CaMIPS1* and *CaMIPS2*” and “*PsMIPS2* and *PsMIPS3*” showed more than 90% similarity.

Moreover, to find out the explanation for the legume *MIPS* gene expansion, the gene duplications were examined in the legume genomes, and a total of 43 duplication pairs were observed. The Ka/Ks value for the duplicated gene pairs was less than 1 for all gene pairs, with a mean value of 0.205 (Supplementary Table S2). These results indicate that in legume species, the *MIPS* genes are constantly evolving through purifying selection.

### Analysis of the cis -regulatory elements in *MIPS*

**Fig. 5:**
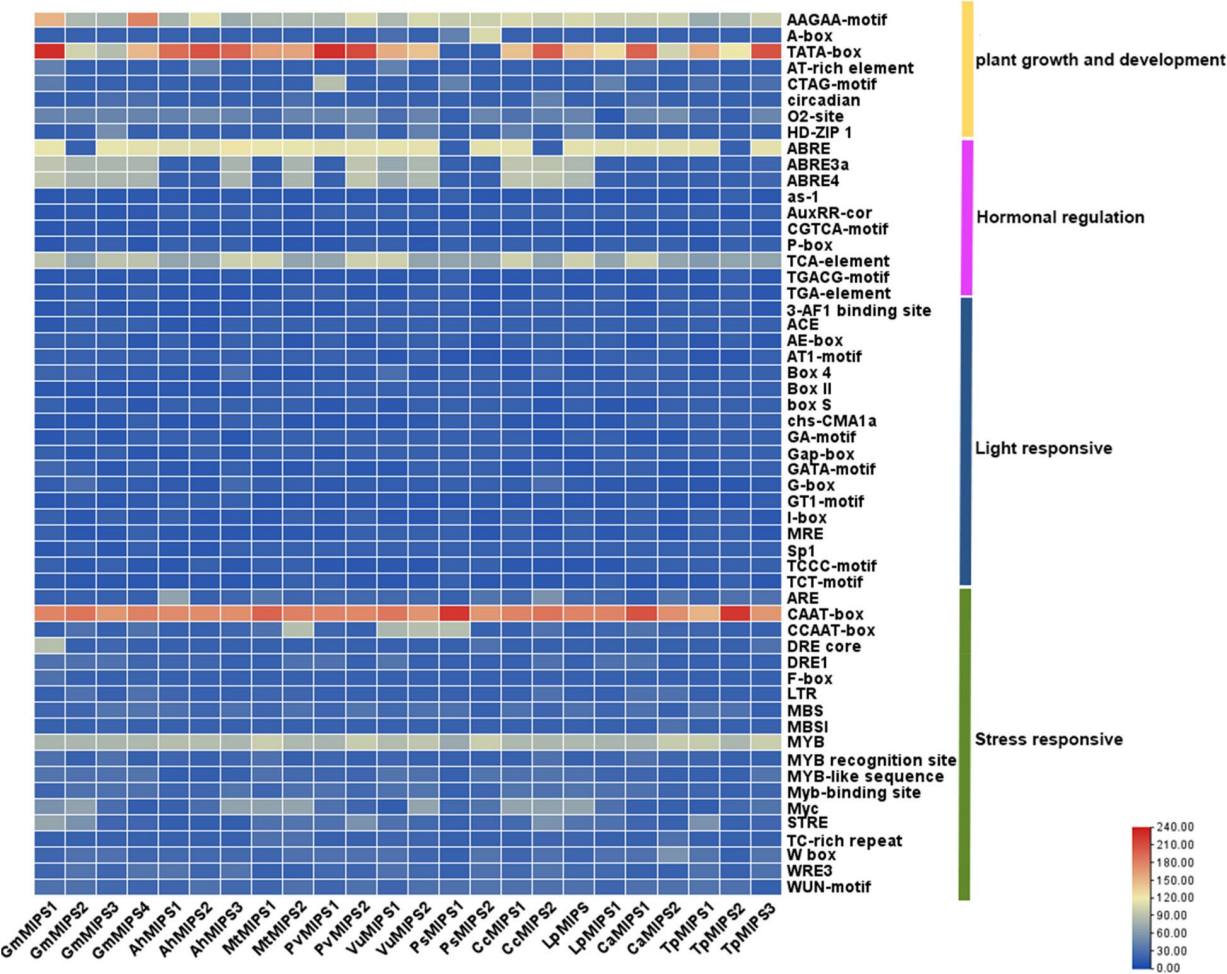
Representation of the number of cis-regulatory elements (CREs) belonging to the following four categories (stress-responsive, hormonal regulation, plant development, and light-responsive) per legume *MIPS* gene as a heat map

To comprehend the likely role of *MIPS* genes in modulating growth and development of legume species, the possible CREs were searched within 2000 bp upstream of *MIPS* genes. A total of 6797 CREs, representing 88 types of CREs, were observed in the 24 *MIPS* genes (Fig. 5). These cis-acting elements were mainly elements related to plant growth (992; 44.22%), elements responsive to abiotic stress (728; 32.45%), followed by elements controlling hormone regulation (655; 15.55%), and elements responsive to light (288; 12.83%). CREs of legume *MIPS* genes involved in plant growth and development include the AAGAA motif, the TATA box, the CAT box, the A box, the AT -rich element and the CTAG motif, which are predominant in legume *MIPS* genes compared to other growth-related elements. Plant hormone-related CREs include TCA element (salicylic acid responsive elements), abscisic acid responsive elements (ABRE), gibberellin responsive elements (P-box, GARE motif), methyl jasmonate responsive elements (CGTCA motif, TGACG motif), auxin responsive elements (AuxRR core, TGA element) and ethylene responsive elements (ERE), which were the third most abundant cis-elements. These results suggest that *MIPS* genes may play an important role in regulating plant growth, and also in various abiotic stress responses.

### GO annotation of *MIPS* gene

**Fig. 6:**
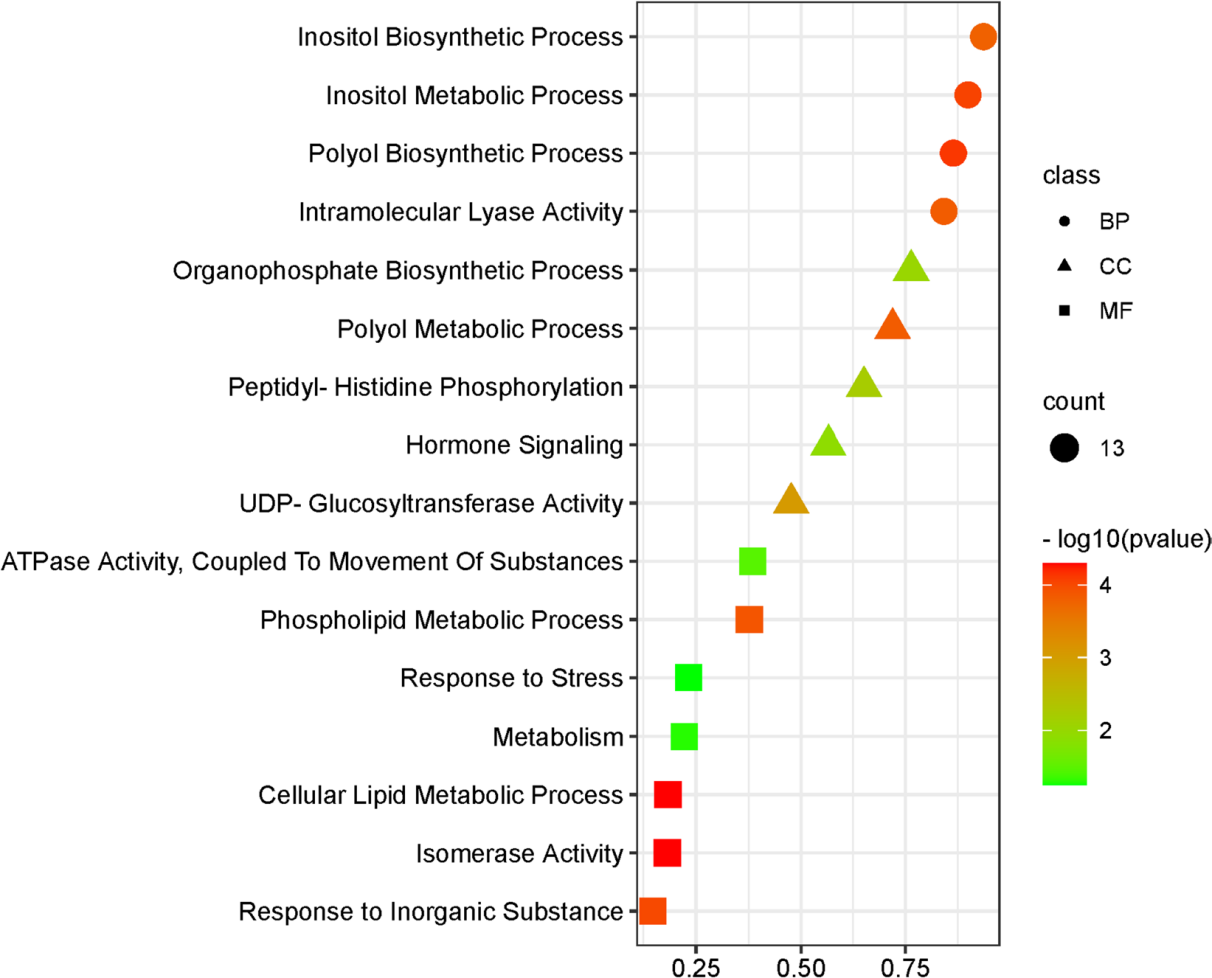
Bubble plot displaying the most enriched GO, BP, CC and MF terms. GO, Gene Ontology; BP, biological process; CC, cellular component; MF, molecule function

GO enrichment was also carried out for the *MIPS* genes to learn more about their roles and the results show that the *MIPS* gene was involved in the synthesis, metabolism and phospholipid metabolism of inositol (phytic acid) (Fig. 6). According to this GO-annotation, they are proven to be crucial for the production of phytic acid and drought tolerance.

### *MIPS* gene expression profile

**Fig. 7:**
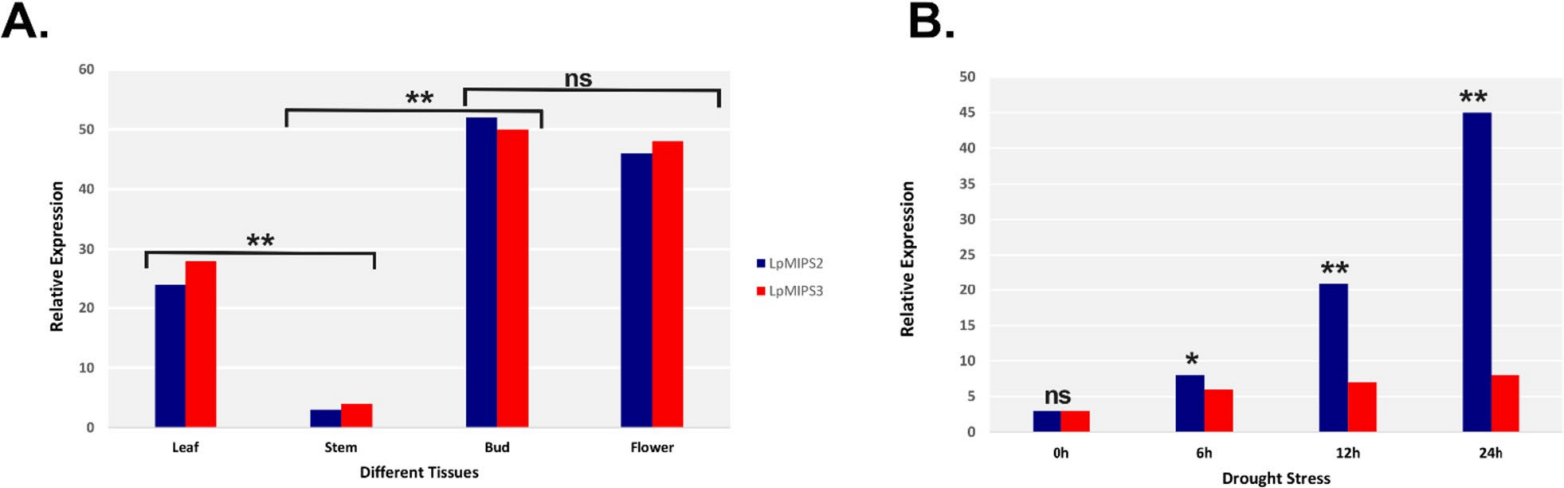
Relative expression level of MIPS in different plant tissue, during drought stress. **A** Relative expression level of LpMIPS2 and LpMIPS3 in different plant tissue. **B** The relative expression level of LpMIPS in leaf tissue at different times (0 h, 6 h, 12 h, and 24 h) treated with 20% PEG and the effect of drought stress. Paired Student’s t-test was used to evaluate the significance of the differences between the samples, with *p*-values of *p < 0.05 and **p < 0.01 being considered significant

To examine the probable biological role of the legume *MIPS* genes, an expression analysis was carried out in *Dolichos bean*. Among leaf, stem, flower bud and flower, the stem tissue had the least expression for *DlMIPS* and flower bud had maximum expression (52.55-fold higher compared to the stem). The *DlMIPS* expression in flower and leaf tissues was 20.68-fold and 14.25-fold higher, respectively compared to that in stem (Fig. 7A).

Expression analysis of *MIPS* gene in dolichos bean plants subjected to drought induction using 20% PEG-6000, was done through RT-qPCR. The expression of the *MIPS* gene under drought stress was normal until 24 h and subsequently up-regulated by 10.65-fold, compared to the control (Fig. 7B).

## Discussion

The L-myo-inositol-1-phosphate synthase, also known as D-myo-inositol-3-phosphate synthase or MIPS (EC 5.5.1.4), is a crucial enzyme in the biosynthesis of inositol and phosphoinositides and is considered an ancient protein, as it is found in a variety of organisms with different evolutionary histories, including fungi, bacteria, animals, and plants [13]. The myo-inositol biosynthetic pathway (MIB) is controlled by MIPS, which converts the precursor glucose-6-phosphate into free myo-inositol. Myo-inositol and its derivatives are vital in plants for several growth and developmental processes, including auxin storage and transport, membrane biogenesis, biosynthesis of phytic acid, signal transduction, etc. [48–50]. The MIPS fulfils the basic physiological functions and is involved in a number of plant stress responses [51–53]. Myo-inositol, synthesised through the MIB pathway, is essential for the formation of phosphoinositide molecules used in evolutionarily well-defined signalling pathways, particularly in the signalling of osmotic stress [54].

The *MIPS* genes have been studied in various plants including, wheat [15], cotton [55], *Brachypodium distachyon* [56], Soybean [7], and Rose [57]. However, the comprehensive identification and characterization of these genes in the legume family has not been done. In this study, a total of 24 genes were identified and described in 10 bean species. In previous studies, 3, 12, and 51 *MIPS* genes were found in *A. thaliana*, *Gossypium hirsutum*, and *Rosa chinensis*, respectively [8, 14, 57]. With the exception of soybean, which contains four *MIPS* genes, the *MIPS* gene number in the rest of the legumes is similar to that in *A. thaliana*, which ranges from two to three. This is most likely due to the tetraploid genome of soybean, which is partially diploidised and may have undergone many duplications [46]. However, the disparity in the number of *MIPS* genes between plant species is not entirely due to genome size. The genome of rice, for example, is only about 430 Mb in size but has 38 *MIPS* genes [58]. *Avena sativa*, on the other hand, has a genome size of up to 11 Gb, but only two *MIPS* genes [59]. Hence, it is crucial to find and describe more *MIPS* genes from different species of plants to understand the several gene expansion methods in various plants.

Phylogenetic analysis revealed that 24 *MIPS* from 10 different legume species are divided into five different clades. Interestingly, all clades had *MIPS* genes from all the species studied, proving that these were conserved and evolved even before the divergence of legume species. Scattered occurrence of the majority of proteins from *Arabidopsis* and legumes, across the subclades, shows that MIPS proteins are functionally conserved in dicots. In addition, most of the legume genes shared *MIPS* exon–intron, motif, and domain arrangements. The amino acid sequence of MIPS has numerous important catalytic domains that are largely conserved across evolutionary lineages, suggesting that they are catalytically active [60]. All the ten species had the four amino acid stretches that are highly conserved, namely LWTANTERY, GWGGNG, GIKPLSIASYN, and INGSPQNTFVPG. These ten motifs were observed in all legume genes except *CaMIPS1*, *TpMIPS2* and *TpMIPS3* and the pattern in which the motifs were arranged was also similar in 21 proteins (Fig. 3C). This suggests that *MIPS* is highly conserved in legumes.

Studies on subcellular localization revealed that most MIPS are positioned in the cytoplasm. Previous studies in various plant species have shown that MIPS is generally found in the cytoplasm but can also be detected in the plasma membrane, endomembrane, and nucleus. These results suggest that MIPS may be associated with signal transduction, membrane trafficking, and gene expression regulation [14, 57].

The genomes of the land plants and gene family sizes have been shaped by genome duplication followed by whole-genome fractionation [61]. Multiple tandem and segmental duplication events also had a key function in the formation of the *MIPS* gene family, as revealed by a gene duplication study of *MIPS* genes in the whole-genomes of *Arabidopsis* and legumes. Recognition of *MIPS* gene orthology may be impacted by the high frequency of gene duplications. Through this study, we could predict 88 types of CREs located on the promoter region of legume *MIPS* genes, which were linked with hormonal regulation, response to abiotic stress, plant development, and photosensitivity. Drought-responsive CREs (ABRE, DRE, ARE, MYC, MYB, and F-box) were predominant in the legume *MIPS* gene when compared to other stress-responsive elements, which points to the fact that legume *MIPS* genes may have a role in stress response in addition to regulating growth and development in plants. Similar studies in other plant species yielded similar results [51, 62, 63]. For example, Gangwar et al. [57] reported that among the various CREs involved in abiotic stress response, CREs related to drought response were the most abundant.

*MIPS* genes are associated with the control of plant growth and development [57]. The wide variation (up to 52.55-fold) of *MIPS* in various plant tissues show that this gene also plays essential roles in growth and development of *Lablab* plants. *MIPS* has also been shown to play a critical role in tolerance to drought [64] and heat [65]. As per the GO analysis, it was observed that *MIPS* genes are mainly involved in the synthesis, metabolism and phospholipid metabolism of inositol (phytic acid). Myoinositol and its derivatives (e.g., pinitol, ononitol, galactinol, and raffinose) play important dual functions as both signals and essential metabolites for osmotic adjustment and ROS scavenging in response to drought stress [64]. In peanut, the *MIPS* gene was identified as a drought-responsive gene that was elevated in transgenic lines that were more drought resistant [66]. Similarly, *MIPS* gene expression was increased during drought stress in *Nicotiana glauca* [67], rose [57], cotton [14], *Arabidopsis* [68], and many others. Overexpressing the *HhMIPS1D* gene of cotton in transgenic *Arabidopsis* improved drought tolerance [14]. Under drought stress, the *MIPS* gene (*OsMIPS1*) splice variants were also increased in rice [65]. Zhai et al. [2016] reported that a myo‐inositol‐1‐phosphate synthase gene, *IbMIPS1* has enhanced the tolerance to salinity and drought and resistance to stem nematodes in transgenic sweet potato [64]. Tan et al. [2013] found that through the ectopic expression of *MfMIPS1* (*Medicago falcata MIPS*) in tobacco, tolerance to drought is increased [69]. In the current study, a substantial increase in the *MIPS* gene expression was detected under the drought stress in *Lablab*, indicating a positive function of the *MIPS* gene for drought resistance in lablab. In addition, we detected drought-responsive elements CREs (i.e. DRE, ABRE, MYC and MYB) in the promoter sequence of *MIPS*, suggesting the involvement of *MIPS* in plant tolerance under low-water conditions.

## Conclusion

We discovered 24 *MIPS* genes belonging to five clades, from ten legume species. Total length of the gene was 6085 bp and gene structure analysis had shown one to nine exons. The presence of multiple components in the promoter sequence, including the growth- and stress-sensitive elements, suggests that *MIPS* plays a critical role in many developmental processes as well as abiotic stresses in legumes. Analysis of *LpMIPS* expression has confirmed the importance of *MIPS* in plant development and drought stress resistance. Current results provide precise data on *MIPS* genes, which could serve as a solid platform for the functional characterization of this important gene family.

### Supplementary Information

**Additional file 1: Table S1.** Detailed information of primers. **Table S2.** Legume MIPS proteins and their related information. **Table S3.** The detailed information of the motifs in MIPS proteins.

## Data Availability

All the results, obtained through this study are included in the article/Supplementary Material. Further clarifications can be received from the corresponding author.

## Abbreviations

aa: Amino acids
BLASTp: Protein-to-protein Basic Local Alignment Search Tool
bp: Base pair
CDD: Conserved Domain Database
cDNA: Complementary DNA
CDS: Coding sequence
CREs: Cis-regulatory elements
G6P: D-glucose-6-phosphate
GO: Gene Ontology
GSDS: Gene Structure Display Server
HMM: Hidden Markov Model
IMP: Myo-inositol monophosphatase
Ins: Myo-inositol
kDa: Kilo Dalton
Ips: Ins polyphosphates
iTOL: Interactive Tree of Life
MEME: Multiple EM for Motif Elicitation
MIPS: *Myo-inositol-1-phosphate synthase*
NCBI: National Center for Biotechnology
NJ: Neighbour-joining
NLS: Nuclear localization signals
PANNZER: Protein ANNotation with Z-scoRE
Pfam: Protein Families
pI: Isoelectric point
PtdIns: Phospholipid phosphatidylinositol
PtdInsP: Phosphoinositide phosphates
qPCR: Quantitative PCR
RIN: RNA integrity number
SMART: Simple Modular Architecture Research Tool
TAIR: The *Arabidopsis* Information Resource
